# Prognostic value of clinical and morphologic findings in patients with type B aortic intramural hematoma

**DOI:** 10.1186/s13019-020-1067-8

**Published:** 2020-03-23

**Authors:** Zilun Li, Chenshu Liu, Ridong Wu, Jian Zhang, Hong Pan, Jinghong Tan, Zhuang Guo, Yingying Guo, Nan Yu, Chen Yao, Guangqi Chang

**Affiliations:** 1 Division of Vascular Surgery, the First Affiliated Hospital of Sun Yat-sen University, Guangzhou, China; 2 National-Guangdong Joint Engineering Laboratory for Diagnosis and Treatment of Vascular Diseases, the First Affiliated Hospital of Sun Yat-sen University, Guangzhou, China; 3 Guangdong Provincial Engineering and Technology Center for Diagnosis and Treatment of Vascular Diseases, the First Affiliated Hospital of Sun Yat-sen University, Guangzhou, China; 4 State Key Laboratory of Ophthalmology, Zhongshan Ophthalmic Center of Sun Yat-Sen University, Guangzhou, China; 5 Zhongshan School of Medicine, Sun Yat-sen University, Guangzhou, China

**Keywords:** Aortic intramural hematoma, Penetrating atherosclerosis ulcer, Chinese population

## Abstract

**Background:**

Aortic intramural hematoma (IMH) is a subset of acute aortic syndrome, and its prognosis may differ between races. This study aimed to study the prognosis of Chinese type B IMH patients and to find out risk factors.

**Methods:**

A total of 71 type B IMH patients with or without penetrating atherosclerosis ulcer (PAU) administrated in our center between September 2013 and October 2017 were retrospectively studied. Both clinical and imaging data were collected and analyzed. The primary end point was aorta-related death, and the secondary end point was progression, which was defined as enlargement of aorta, increased aortic wall thickness, and aortic dissection or aneurysm formation. Kaplan-Meier survival analysis and Cox regression analysis were used for prognostic analysis.

**Results:**

Among these 71 patients, 21 had simple type B IMH, when 50 had type B IMH in association with PAU. Twenty-five patients received optimal medical therapy (OMT) alone, while 46 patients received surgery and OMT. The mean follow-up time was 27.5 ± 13.5 months. For type B IMH patients, association with PAU indicated poor prognosis and required more intensive management (HR = 16.68, 1.96~141.87), while maximum aortic diameter (MAD) was an independent risk factor (HR = 1.096, 1.016~1.182). For patients with PAU-IMH, MAD was an independent risk factor (HR = 1.04, 1.021~1.194), while surgical treatment was independent protective factor (HR = 0.172, 0.042~0.696).

**Conclusion:**

Association with PAU and MAD were independent risk factors for type B IMH patients. Surgery may improve the outcomes for type B IMH in association with PAU.

## Background

Aortic intramural hematoma (IMH) is a subset of acute aortic syndrome (AAS), and is defined as crescentic or circumferential thickening of the aortic wall without any entry point by imaging techniques [1, 2]. The epidemiology of IMH varies among different regions world-wide. 31.7% of patients were diagnosed as IMH relative to typical aortic dissection in Japan/Korea population, compared with 10.9% in North America (NA) /Europe. This difference might be partially due the diagnosis criteria in different countries. Of note, significant difference of mortality rate between NA/Europe and Japan/Korea was also observed [3]. These differences suggest that the morbidity and mortality of IMH may be associated with race. Yet, studies from mainland China are scarce. Thus, this study aimed to demonstrate characteristics of Chinese patients with type B IMH, and to find out prognostic factors based on our single center experience.

## Methods

### Patients

From September 2013 to October 2017, 71 consecutive type B IMH patients administered or referral to our center were retrospectively studied. Type B IMH associated with penetrating atherosclerotic ulcer (PAU) was not excluded. Clinical data including demographic characteristics and potential prognostic factors were extracted.

### Imaging techniques

**Fig. 1:**
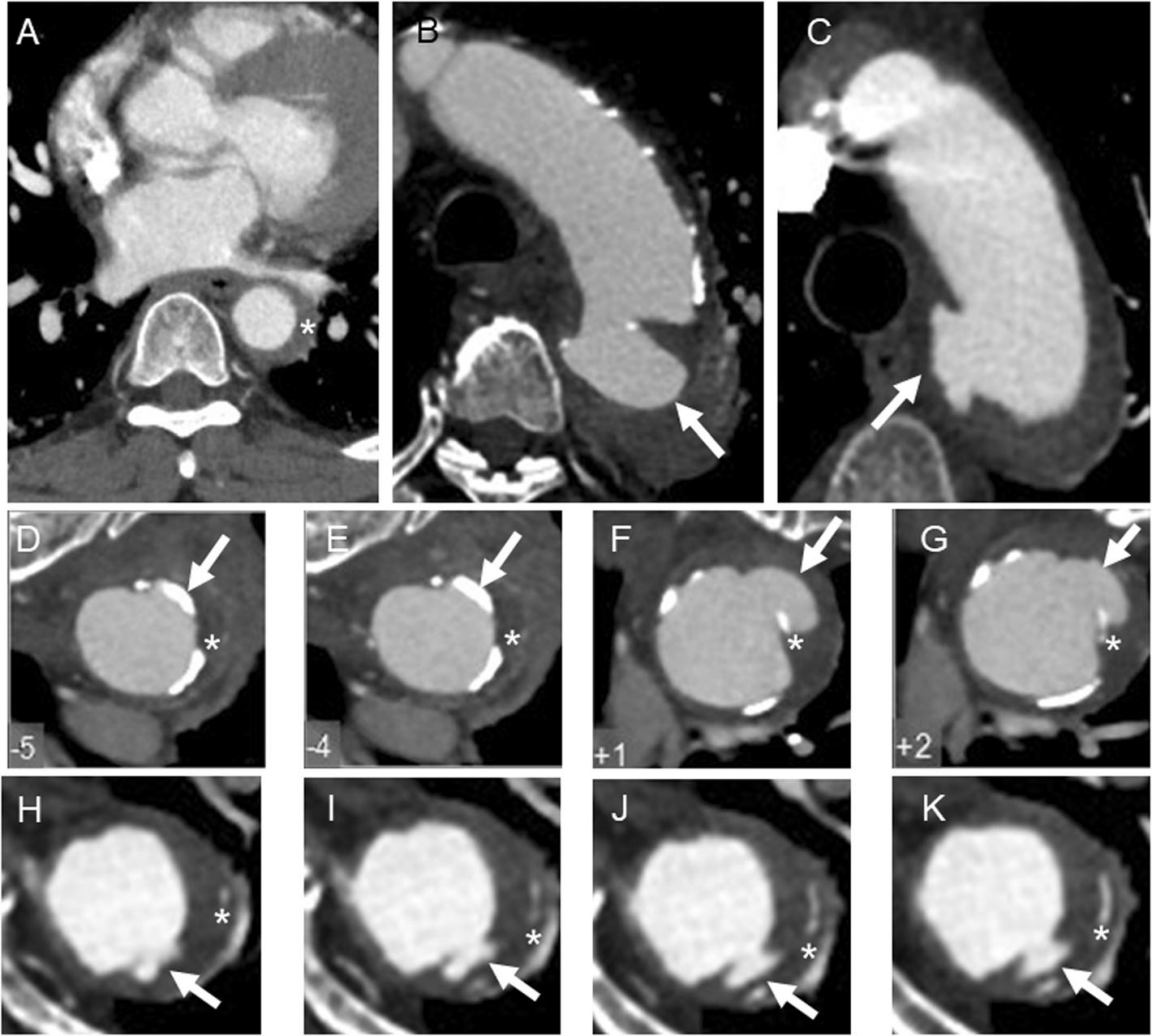
**a** Typical intramural hematoma was defined as crescentic or circumferential uniform thickening of the aortic wall (> 5 mm) without any intimal tear (Asterisk indicated). **b** & **c** Penetrating ulcer was defined as a focal disruption in the arterial intima (Arrow indicated). **d** - **g** For differentiation of PAU and dissection, the calcification of intima was used as auxiliary line to locate the intima. As shown in **d** and **e**, the frontier of projection (Arrow indicated) was calcified intima (Asterisk indicated), which protruded into the media on the following cross sections (**f** and **g**). **h** - **k** The projection (Arrow indicated) was located inside the calcified intima (Asterisk indicated), which is considered as rupture secondary to type B IMH

Computed tomography angiography (CTA) was performed for all patients within 24 h after administration. IMH was defined as crescentic or circumferential thickening of the aortic wall (> 5 mm) without any intimal tear, comparing with aortic dissection (AD). PAU was considered as a focal disruption in the arterial intima and elastic lamina protruding into the media [4], whereas IMH associated with PAU was termed as PAU-IMH. Further differential diagnosis between PAU-IMH and rupture secondary to IMH was made with the presence or absence of intimal calcification [5] (Fig. 1). Maximum diameter of aorta and maximum thickening were measured, and PAU was recorded [6–8]. All CTA data were independently reviewed by two physicians, and discrepancy was settled with discussion.

### Management

Optimal medical therapy (OMT) consisted of betablockers and/or other antihypertensive drugs. Patients with type B IMH only, stable hemodynamic and no indication for open surgery were referred to OMT and reexamination of CTA was performed 2 weeks later for further evaluation [9, 10]. For PAU-IMH, surgery was considered if the ulcer had a width > 15 mm or depth > 10 mm, or other indications [11, 12].

### Follow-up protocol

Phone call follow-up was conducted quarterly, while CTA was performed at 6 months, 12 months and yearly thereafter. Within a mean follow-up of 26.5 ± 13.0 months, 7 patients (9.8%) were lost. All patients received at least 2 CTA studies during follow-up. The primary endpoint was aorta-related death, and the secondary endpoint was progression, which was defined as enlargement of aorta> 5 mm, increased aortic wall thickness > 5 mm, and AD or aneurysm formation during follow-up [13].

### Statistical analysis

Continuous values are expressed as mean ± SD and *t* test was used for comparison. Categorical variables were expressed as frequencies and percentages and were compared with *χ*2 or Fisher’s exact-test. Survival curves were generated via the Kaplan–Meier method with significant differences assessed for time-to-event data using log-rank tests. Cox regression models were constructed to identify factors associated with prognosis and survival rates. *P* value < 0.05 was considered significant. The analyses were performed using SPSS version 24.0 (IBM SPSS, Chicago, Illinois).

## Results

### Patient characteristics

Among the 71 patients, 21 had simple type B IMH, while 50 had PAU-IMH (30% vs. 70%, Table 1). The mean age of 71 patients was 62.6 ± 10.6, and dominantly 60 of them (84.5%) were male. Patients with type B PAU-IMH were significantly older than those with simple type B IMH (65.2 ± 9.2 vs. 56.3 ± 11.4, *P* < 0.001). Hypertension (54%) and smoking (54%) were the most common risk factor, followed by diabetes mellitus (13%), hyperlipemia (3%). Coronary (7%) and congenital (4%) heart disease were also observed in both groups. Connective tissue diseases such as Marfan syndrome were not observed (data not shown). For all patients, 9 patients had a history of aortic disease (1 AD, 8 Aortic Aneurysm), whereas 6 patients had a history of surgery (1 open repair for AA, 2 endovascular repair for AA, 1 endovascular repair for AD, and 2 others). No significant difference of history was observed between the two groups.

**Table 1:** Patients’ Basic Characteristics

Category	IMH (*n* = 21)	PAU-IMH (*n* = 50)	*P* value
Background			
Age, y (mean ± SD)	56.3 ± 11.4	65.2 ± 9.2	< 0.001
Male/Female	18/3	42/8	> 0.99
Smoking, n(%)	11(52.4%)	27(54.0%)	0.901
Hypertension	12(57.1%)	26(52.0%)	0.692
Coronary heart disease	0	5(10.0%)	0.312
Congenital heart disease	1(4.8%)	2(4%)	> 0.99
Diabetes	1(4.8%)	8(16.0%)	0.194
Hyperlipemia	1(4.8%)	1(2%)	0.507
Pregnancy	0	1(2%)	> 0.99
History			
History of AoD^a^	2(9.5%)	7(14%)	0.605
History of any surgery	0	6(12%)	0.170

^a^*AoD* Aortic Disease

### Initial symptoms and comorbidities

As shown in Table 2, the most common symptom was chest pain (54%) and back pain (54%), which was followed by abdominal pain (23%). Chest distress (11%), dyspnea (8%), syncope (1%) and hemothorax (1%) were also observed. Among all the initial symptoms, more type B PAU-IMH patients showed no initial relevant pain during administration (26% vs. 0, *P* = 0.007). In these 13 pain free patients, 3 had chest distress as initial symptom, 1 had syncope, 1 had spinal cord ischemia, while the remaining 8 had no symptoms.

**Table 2:** Initial Symptoms

Category	IMH (*n* = 21)	PAU-IMH (*n* = 50)	*P* value
Associate pain, n(%)			
Chest pain	14(66.7%)	24(48%)	0.196
Abdominal pain	4(19%)	12(24%)	0.649
Back pain	12(57.1%)	26(52%)	0.692
Waist pain	4(19%)	9(18%)	> 0.99
None	0	13(26%)	0.007
Chest distress	4(19.0%)	4(8.0%)	0.179
Aortic regurgitation	0	0	
Pericardial effusion	1(1.4%)	0	0.296
Myocardial ischemia	1(1.4%)	4(8.0%)	> 0.99
Dyspnea	2(9.5%)	4(8.0%)	> 0.99
Spinal cord ischemia	1(4.8%)	1(2.0%)	0.507
Syncope	0	1(2.0%)	> 0.99
Hemothorax	0	1(2.0%)	> 0.99
No symptom	0	7(14.0%)	0.096

### Treatment

**Fig. 2:**
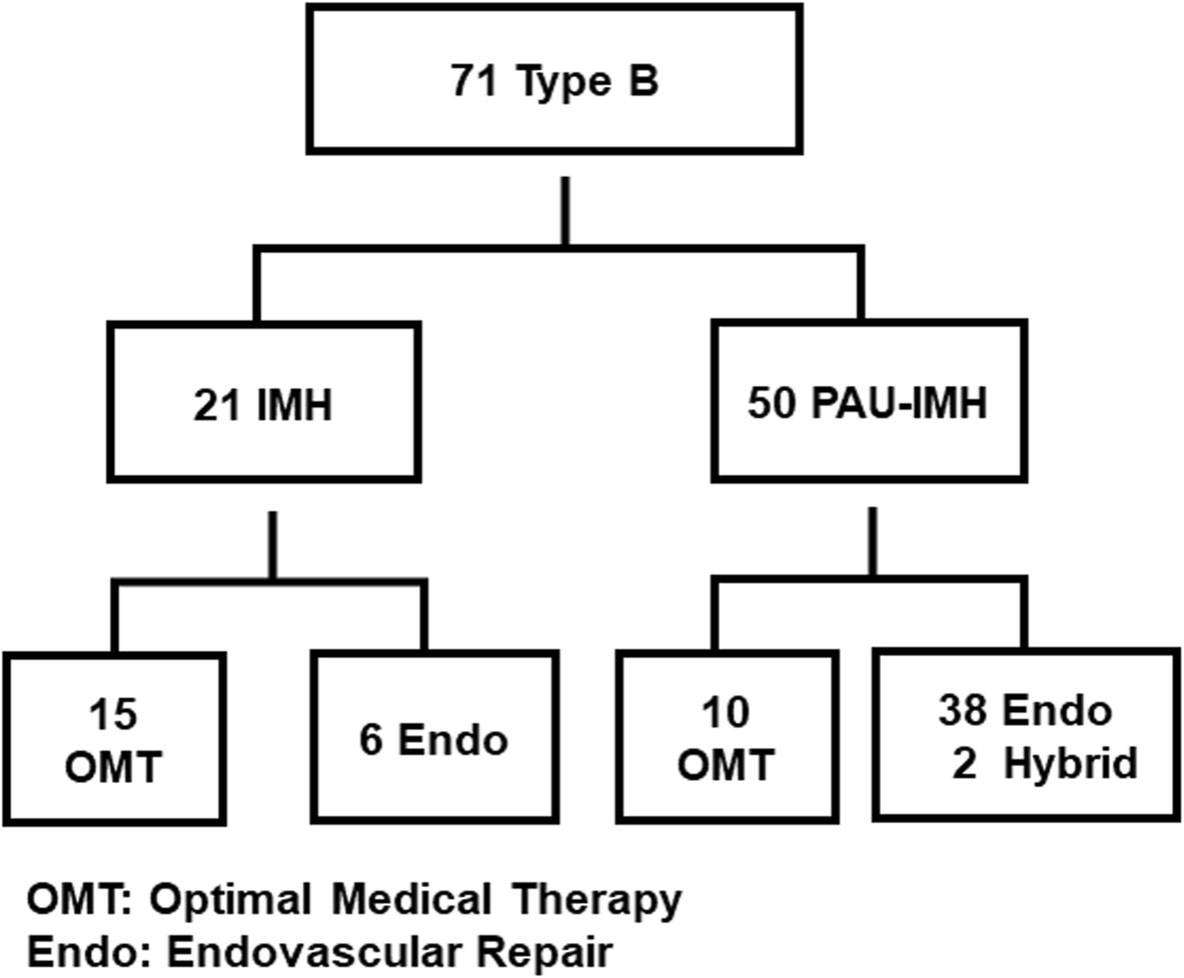
Flowchart of patients’ treatment

Among all the 71 type B IMH patients, 21 had simple type B IMH, while 50 had type B PAU-IMH (Fig. 2). In 50 patients with type B PAU-IMH, 38 patients received endovascular repair, and 2 underwent hybrid surgery, considering that the ulcer had a width > 15 mm or depth > 10 mm or other indications [11, 12]. For simple type B IMH, 6 patients received endovascular repair for refractory pain in 3, progression to aortic dissection in 2 and aneurysm in 1, while the rest were managed with OMT.

**Fig. 3:**
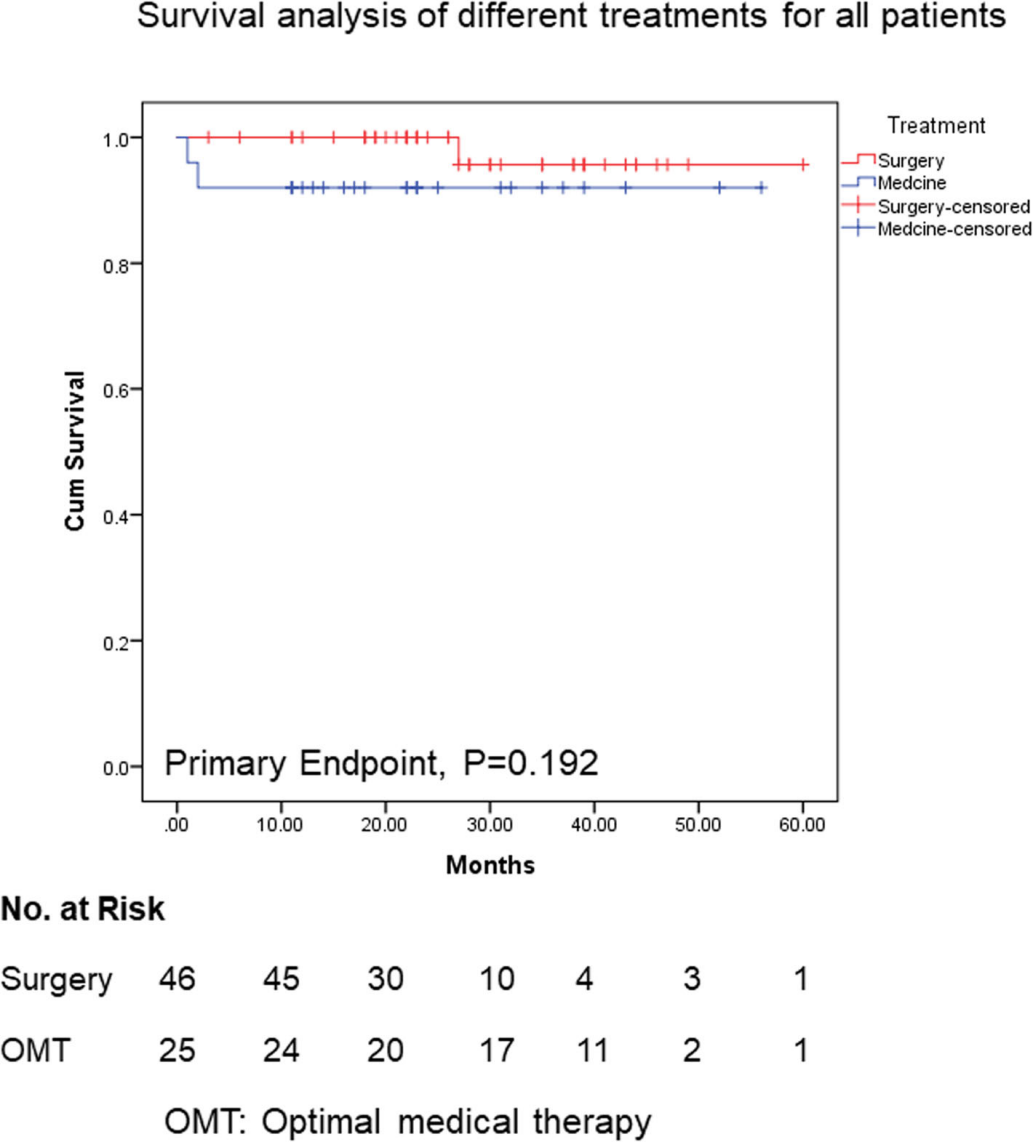
Kaplan-Meier survival curve for all type B IMH patients, surgery vs optimal medical therapy (OMT)

**Fig. 4:**
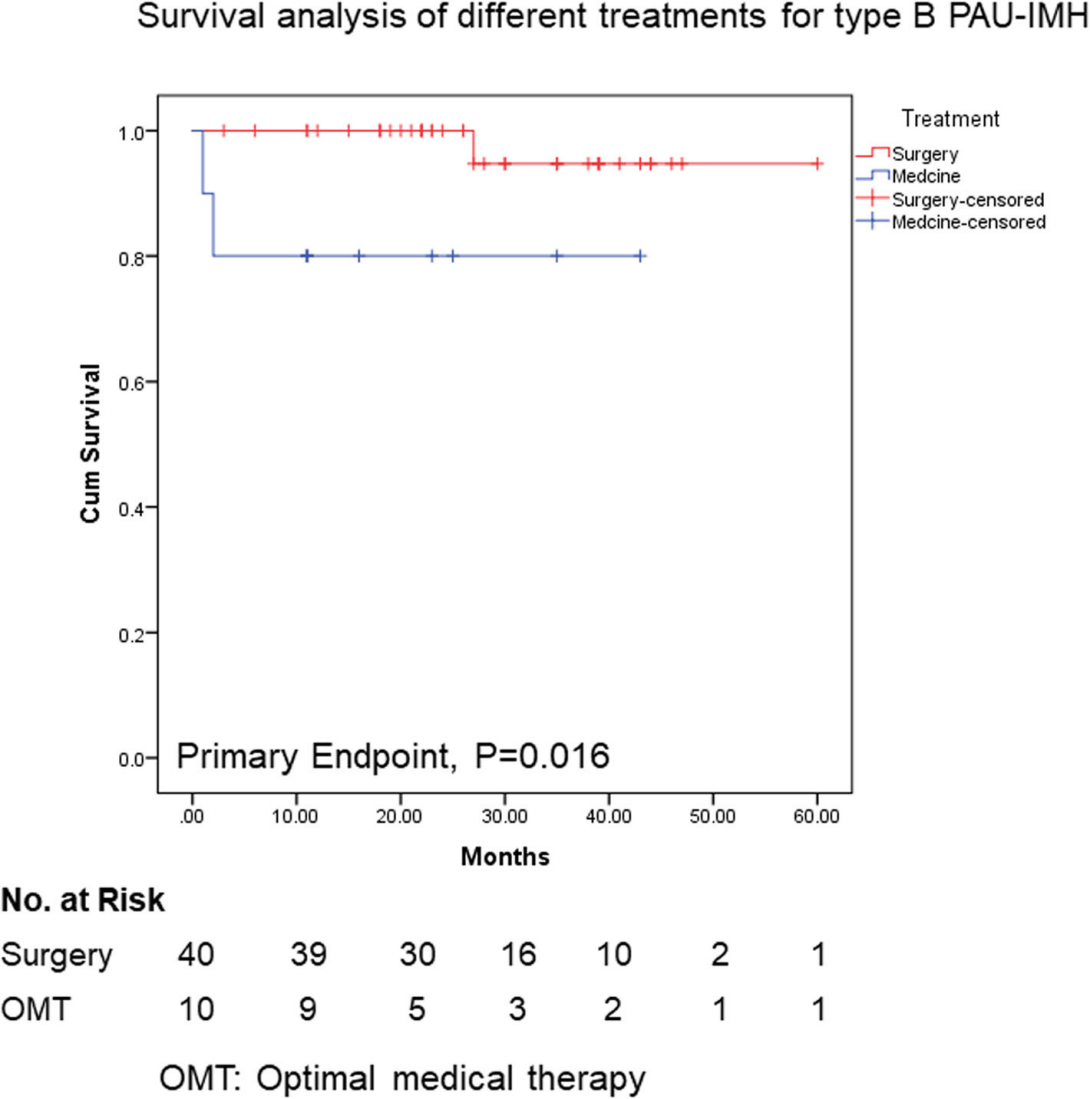
Kaplan-Meier survival curve for type B PAU-IMH patients, surgery vs optimal medical therapy (OMT)

As optimal therapeutic strategy for type B IMH remains debated [14, 15], we compared the outcomes of surgery combined with OMT and OMT only in all patients. As shown in Fig. 3, Kaplan–Meier survival analysis of primary endpoint illustrated no significant difference in both groups (*P* = 0.192, Fig. 3). In the IMH group, there was no difference of prognosis between OMT and surgery treatment (data not shown). We next compared two treatments in type B PAU-IMH patients, and survival analysis showed that surgery improved the prognosis as for primary endpoint (*P* = 0.016, Fig. 4). This result was in consistence with one previous study [11].

### Prognosis

In order to study possible risk factors for type B IMH, we performed multivariable analysis in our cohort. The Cox regression models showed that for all patients in our cohort (Table 3), association with PAU (HR = 16.68, 1.960~141.87) and MAD (HR = 1.096, 1.016~1.182) were independent risk factors, whereas surgery was an independent protective factor (HR = 0.123, 0.032~0.478). Next, we studied type B IMH and PAU-IMH patients separately. The simple type B IMH group contained 21 patients and was too small to bear multivariable analysis. For patients with type B PAU-IMH (Table 4), the model showed that MAD was an independent risk factor (HR = 1.04, 1.021~1.194), while surgical intervention was an independent protective factor (HR = 0.172, 0.042~0.696).

**Table 3:** Cox Regression Analysis of Patients Progression

Variable	Progression (*n* = 16)	Regression/Stable (*n* = 55)	HR	95% CI		*P*
Age	63.3 ± 11.3	62.3 ± 10.5	0.993	0.929	1.062	0.842
Surgery	9(56.3%)	37(67.3%)	0.123	0.032	0.478	0.002
PAU	15(93.8%)	35(63.6%)	16.68	1.960	141.870	0.010
MAD	44.3 ± 8.8	40.0 ± 7.7	1.096	1.016	1.182	0.017
MHT	13.2 ± 6.9	12.7 ± 6.8	0.931	0.851	1.018	0.114

*MAD* Maximum Aortic Diameter, *MHT* Maximum Hematoma Thickness

**Table 4:** Cox Regression Analysis of PAU-IMH Patients Progression

Variable	Progression (*n* = 15)	Regression/Stable (*n* = 35)	HR	95% CI		*P*
Age	62.6 ± 11.3	66.3 ± 8.1	0.965	0.900	1.034	0.312
Surgery	9(60.0%)	31(88.6%)	0.172	0.042	0.696	0.014
MAD	44.3 ± 9.1	41.4 ± 9.0	1.04	1.021	1.194	0.014
MHT	13.1 ± 7.1	13.7 ± 8.0	0.929	0.846	1.020	0.121

*MAD* Maximum Aortic Diameter, *MHT* Maximum Hematoma Thickness

## Discussion

IMH is one type of AAS, which is probably due to vasa vasorum rupture [16], while some studies claimed that IMH should be referred as thrombosed-type aorta dissection [17, 18]. Although the International Registry of Acute Aortic Dissections (IRAD) illustrated similar prognosis of AD and IMH [1, 19, 20], some other researchers reported better prognosis and survival rate for IMH when compared with typical AD [1, 21, 22]. Nevertheless, IMH may progress to AD, aneurysm formation, or even death. Thus, it is important to study the prognosis of IMH with different treatment strategies and possible risk factors.

Firstly, we analyzed the impact of treatment on prognosis. Though surgery did not illustrate significant difference in Kaplan–Meier survival analysis for all type B IMH patients, it showed a better prognosis in type B PAU-IMH patients when compared with OMT (*P* = 0.016). Furthermore, Cox regression analysis showed that surgery presented as an independent protective factor for all type B IMH patients, which might be a confounding bias, considering a large portion of type B PAU-IMH patients (70.2%) in this study. Thus, the type B PAU-IMH patients were analyzed separately, and surgery was confirmed as an independent protective factor as adjusted with age, MAD, and maximum hematoma thickness (MHT). Therefore, patients with type B PAU-IMH may benefit from surgery. Although ACCF-TAD Guidelines 2010 and JCS-AD Guidelines 2011 did not give recommendation on patients with type B PAU-IMH, ESC-AD Guidelines 2014 recommended open surgery or endovascular repair [23]. According to our data, patients with type B PAU-IMH should be referred to surgery more aggressively.

Previously reported risk factors, like PAU, MAD, and MHT [6, 13, 24, 25], were further used to explore prognostic risk factors of type B IMH. For our study, association with PAU was an independent risk factor for type B IMH patients (HR = 16.68, 1.960~141.87) as well as MAD (HR = 1.096, 1.016~1.182). Furthermore, MAD remained an independent risk factor for type B PAU-IMH patients (HR = 1.04, 1.021~1.194). However, due to the small sample size, we failed to set up a cut-off value for MAD, which would be addressed in our future study.

## Conclusion

For Chinese type B IMH patients, association with PAU and MAD were independent risk factors. And for type B PAU-IMH patients, MAD was an independent risk factor when surgery was an independent protective factor.

### Limitations

First, our study was a retrospectively observational study with a short-term follow-up, which may lead to potential bias and results should be considered as preliminary. Second, the sample size was rather small due to the low morbidity rate of type B IMH, which hindered further analysis with simple type B IMH patients and MAD.

## Data Availability

The datasets used and/or analyzed during the current study are available from the corresponding author on reasonable request.

## Abbreviations

AA: Aortic Aneurysm
AAS: Acute Aortic Syndrome
ACCF-TAD: American College of Cardiology Foundation-Guidelines for the Diagnosis and Management of Patients With Thoracic Aortic Disease
AD: Aortic Dissection
CTA: Computed Tomography Angiography
ESC-AD: European Society of Cardiology-Guidelines for diagnosis and treatment of aortic diseases
IMH: Intramural Hematoma
JCS-AD: Japanese Circulation Society-Guidelines for diagnosis and treatment of Aortic Aneurysm and Aortic Dissection
MAD: Maximum Aortic Diameter
OMT: Optimal Medical Therapy
PAU: Penetrating Atherosclerosis Ulcer
